# The Burden, Risk Factor Analysis, and Prediction of Low Back Pain in China From 1990 to 2021

**DOI:** 10.1155/prm/6863134

**Published:** 2026-09-16

**Authors:** Antao Lin, Yuanye Ma, Chengwei Zhang, Wenke Yang, Shuo Han, Hao Zhang, Zhiming Liu, Shuai Tang, Wenhua Gong, Xuexiao Ma

**Affiliations:** ^1^ Department of Spinal Surgery, The Affiliated Hospital of Qingdao University, Qingdao, Shandong, China, qdu.edu.cn; ^2^ Department of Clinical Medicine, Qingdao University, Qingdao, Shandong, China, qdu.edu.cn; ^3^ Department of Medical Imaging, The Affiliated Hospital of Qingdao University, Qingdao, Shandong, China, qdu.edu.cn; ^4^ Department of Orthodontics, The Affiliated Hospital of Qingdao University, Qingdao, Shandong, China, qdu.edu.cn

**Keywords:** Global Burden of Disease study, incidence, low back pain, prevalence, years lived with disability

## Abstract

**Introduction:**

The burden of low back pain (LBP) in China poses a substantial public health challenge. This study aimed to analyze longitudinal trends in the burden of LBP and its associated risk factors based on data from the Global Burden of Disease (GBD) 2021 study and to project future trends.

**Methods:**

Epidemiological data for LBP in China and globally were retrieved from the GBD 2021 database. Disease burden was characterized using summary tables and age‐dependent line graphs. Joinpoint regression analyses were used to investigate temporal trends in incidence rates and risk factors in China. The periods between identified joinpoints were analyzed by decomposition analysis to investigate the drivers of change. A log‐linear Poisson age‐period‐cohort model was fitted to age‐period‐specific incident counts, with the corresponding population exposure included as an offset, to estimate age, period, and birth cohort effects on LBP incidence. Finally, a Bayesian age‐period‐cohort (BAPC) model was used to predict the trend of the age‐standardized incidence rate of LBP in China.

**Results:**

From 1990 to 2021, the number of prevalent cases and total years lived with disability (YLDs) due to LBP increased both globally and in China, while the age‐standardized prevalence rate (ASPR) and age‐standardized YLD rate (ASYR) decreased. The joinpoint regression analysis of incidence rates identified four significant turning points in China, which defined five periods over the past 30 years. Decomposition analyses identified aging as a key driver of increased incident cases and showed the escalating impact of population growth. Occupational ergonomic factors were the main contributors to ASYR, while the impact of high BMI escalated significantly, reaching an annual percentage change (APC) of 3.65 in 2008–2021. Smoking was the most prominent factor for middle‐aged and older men. BAPC posterior means showed a marginal increase in incidence rates among men and a slight decrease among women; however, the 95% credible intervals widened substantially and overlapped by 2036. These findings should be interpreted as uncertain posterior mean patterns rather than firm future trends.

**Conclusion:**

The absolute burden of LBP in China has increased over the past 30 years in the context of demographic shifts, while age‐standardized rates have trended downward. The timing of this decline partly coincided with the implementation of key national health policies, raising the hypothesis that these policies may have contributed to the observed population‐level changes. However, temporal alignment in this descriptive ecological analysis does not establish causality or demonstrate policy effectiveness. To address the continuing burden, occupational health, weight management, and tobacco control remain important areas for prevention.

## 1. Introduction

Globally, low back pain (LBP) has emerged as a serious public health problem, characterized by an increase in years lived with disability (YLDs) and serving as a significant contributor to workforce attrition. LBP not only severely affects an individual’s quality of life but also has far‐reaching socioeconomic implications.

LBP is defined as pain occurring between the inferior borders of the lower ribs and the gluteal crease [1]. It is usually accompanied by pain in one or both legs with associated neurological symptoms. LBP is categorized as a symptom rather than a distinct disease entity. Its etiology is multifaceted, involving a complex interplay of biophysical, psychological, and social factors that collectively dictate the clinical trajectory and contribute to substantial healthcare expenditures [2].

According to the Global Burden of Disease (GBD) 2021 study, LBP remains the leading cause of the global burden of disability [3]. Furthermore, current projections indicate that both the aggregate burden and the associated healthcare expenditures are poised to escalate significantly over the coming decades [2]. In 2018, The Lancet published a three‐part series on the definition of LBP, the best evidence‐based treatments, and future research directions. Importantly, the role of advice and education in self‐management, physical, and psychological interventions was emphasized, particularly as first‐line treatments for LBP [2, 4, 5]. Regarding risk factors for LBP, the GBD 2021 Low Back Pain Collaborators reported that two‐fifths of the burden is attributable to modifiable risk factors [3]. Therefore, it is of great significance to analyze the burden and risk factors of LBP in the population.

The decade 2020–2030 has been designated as the United Nations Decade of Healthy Aging, and this initiative provides a strong platform to strengthen national, regional, and global health initiatives to reduce the burden of LBP through public awareness campaigns and the provision of advice [3]. According to the 2020 census in China, the proportion of Chinese seniors aged 65 and over is approaching 14%. China has become one of the fastest‐aging countries, with more people aged 65 and over than in any other country, and the proportion of older people in the country’s total population is expected to reach about 25% by 2050 [6]. Given that LBP typically occurs in the elderly population, the health needs of China’s older adults pose a serious and unprecedented challenge to the country’s health and social care systems. Over the past 20 years, China has implemented a series of health reforms and chronic disease policies with potential relevance to the prevention and management of LBP. In 2009, the government embarked on a major healthcare reform to provide equal access to healthcare services for all citizens. The Healthy China 2030 Strategy represents a significant policy shift from the 2009 reforms, formalizing the integration of health into all aspects of national policy. It frames population health improvement not only as a social imperative but as a fundamental driver for long‐term economic stability and growth [7]. In this context, studies related to the changing trends and variable risk factors of the burden of LBP in China are of great significance for the management of the burden of LBP in China and even globally.

This study analyzes the burden and modifiable risk factors of LBP in China by age, sex, and period using estimates from the GBD 2021 study. Rather than introducing a new estimation framework, we aim to provide a comprehensive characterization of temporal trends in LBP burden, risk factor patterns, and demographic drivers in China. By integrating joinpoint regression, decomposition analysis, age‐period‐cohort (APC) analysis, and Bayesian age‐period‐cohort (BAPC) forecasting, this study offers additional epidemiological insights into how population aging, growth, and modifiable exposures have shaped the changing burden of LBP in China.

## 2. Methods

### 2.1. Data Source

The epidemiological estimates used in this study were obtained from the Global Burden of Disease Study 2021 (GBD 2021), coordinated by the Institute for Health Metrics and Evaluation (IHME). The data are publicly accessible through the Global Health Data Exchange (GHDx) GBD 2021 data portal (https://ghdx.healthdata.org/gbd-2021) and were retrieved using the GBD Results Tool (https://vizhub.healthdata.org/gbd-results/). No additional ethical approval was required because all data used in this study were publicly available and contained no personally identifiable information. These data were identified via a systematic review of the electronic databases Ovid Medline, Embase, and CINAHL, supplemented by opportunistic searches; reviews of government and international organization websites, published reports, demographic and health surveys, and datasets contributed by GBD collaborators [3].

We extracted the following indicators related to LBP for China and globally, from 1990 to 2021: number of prevalent cases, age‐standardized prevalence rate (ASPR) per 100,000 population, number of incident cases, age‐standardized incidence rate (ASIR) per 100,000 population, YLDs, age‐standardized YLD rate (ASYR) per 100,000 population, and YLDs attributable to three modifiable risk factors: occupational ergonomic factors, smoking, and high body mass index (BMI). Details are provided in Table 1. Data were stratified by sex (male, female, both), age group (5‐year intervals from 5 to 9 years to 95+ years), and year (1990–2021). All estimates were accompanied by 95% uncertainty intervals (UIs), reflecting uncertainty due to sampling, model specification, and data sparsity.

**TABLE 1:** Definitions and calculation methods of core indicators.

Indicator	Abbreviation	Definition	Unit	Age‐standardized	GBD source/model
Prevalence cases	—	Total number of existing cases of low back pain in a given population during a specific time period.	Count (persons)	No	Derived from DisMod‐MR estimated prevalence × population
Prevalence rate	—	Proportion of the population affected by low back pain at a given time, per 100,000 persons.	Per 100,000	No	Direct output from DisMod‐MR
Age‐standardized prevalence rate	ASPR	Prevalence rate adjusted to a standard population age structure (e.g., GBD global standard population) to eliminate age structure differences.	Per 100,000	Yes	Direct standardization using age‐specific prevalence from DisMod‐MR
Incident cases	—	Number of new cases of low back pain occurring within a specific time period (usually 1 year).	Count (persons)	No	Derived from DisMod‐MR estimated incidence × population
Incident rate	—	Number of new cases of low back pain per 100,000 population within a specific time period.	Per 100,000	No	Direct output from DisMod‐MR
Age‐standardized incidence rate	ASIR	Incidence rate adjusted to a standard population age structure.	Per 100,000	Yes	Direct standardization using age‐specific incidence from DisMod‐MR
Years lived with disability	YLDs	Healthy life years lost due to nonfatal health conditions (low back pain).	Person‐years	No	Prevalence cases × disability weight (from GBD surveys/expert assessments)
Age‐standardized YLD rate	ASYR	YLDs per 100,000 population, adjusted to a standard population age structure.	Per 100,000	Yes	Direct standardization based on age‐specific YLDs
Disability‐adjusted life years	DALYs	Total healthy life years lost due to premature death and disability. Note: For low back pain, YLLs = 0 as it is nonfatal.	Person‐years	No	YLDs + YLLs (YLLs = 0 for low back pain)

The data on the number of incident cases, incidence rate, and ASIR used in this study were obtained directly from the GBD Results Tool available through the GHDx and are official epidemiological indicators released by GBD 2021. For the APC analysis, the data were arranged as age‐period‐specific incident counts and corresponding population exposures. Age‐specific incidence rates are rates within individual age strata and are distinct from ASIR, which is a weighted summary across age groups; ASIR was not used as the APC outcome. The Bayesian meta‐regression tool (DisMod‐MR 2.1) was used to generate all prevalence and incidence estimates by age, sex, location, and year. Next, LBP was classified into six levels according to its severity and whether it was accompanied by leg pain; two of these levels corresponded to health states with or without leg pain, and each health state was linked to a disability weight. YLDs were calculated by multiplying the disability weights for each health state by the corresponding age‐, sex‐, location‐, and year‐specific prevalence rates, and the resulting disability rates and burdens were provided.

In the current GBD data, no deaths were attributed to LBP. Accordingly, the estimates for DALYs and YLDs were equivalent, with the burden of disability reported as YLDs. Data on modifiable risk factors for LBP, including occupational ergonomic exposures, smoking, and high BMI, were obtained primarily from the International Labor Organization, nationally representative household surveys, and systematic reviews [8]. The GBD risk factor attribution follows the comparative risk assessment framework, which estimates the population‐attributable fraction based on theoretical minimum risk exposure levels. These estimates reflect associations under idealized assumptions and should not be interpreted as direct causal effect sizes in real‐world interventions.

### 2.2. Case Definition

LBP is defined as pain in the posterior part of the body from the lower edge of the 12th rib to the subgluteal crease, with or without pain in one or both of the lower extremities, lasting at least 1 day [9].

According to the findings of the GBD 2021 study, the modifiable GBD risk factors for LBP remain occupational ergonomic factors, smoking, and high BMI [3]. Occupational ergonomic factors were used to represent lifting, forceful movements, awkward postures, vibration, etc., as specific data on these factors were not available for each country/region [10]. High BMI was defined as a value exceeding the theoretical minimum risk exposure level (i.e., the BMI range associated with the lowest risk, namely 20–25 kg/m^2^) [11]. It is well established that for people with high BMI, the mechanical forces exerted on lumbar spine structures are greater under both normal and weight‐bearing conditions, resulting in a higher risk of LBP [12]. In addition, previous research has shown a potential association between smoking and disc degeneration [13].

### 2.3. Statistical Analysis

Joinpoint regression software (Version 5.1.0.0) was used to analyze temporal trends in the ASIR of LBP and ASYR attributable to various risk factors in China from 1990 to 2021. In addition, the annual percentage change (APC) was calculated for each period. The model specifications were as follows: 1. Maximum number of joinpoints: Set to 5 (allowing up to 6 segments) to identify key time points of trend changes; 2. Model form: A log‐linear model was applied, assuming that the log of the rate changes linearly over time; 3. Permutation test: The Monte Carlo permutation method was used to test the significance of joinpoints, with 4499 permutations; 4. Significance level: A significance threshold of *α* = 0.05 was used to determine statistically significant changes in trends; 5. Point estimate type: The point estimate (value) of the age‐standardized rate was used as the input to the model.

Das Gupta‐based decomposition analysis can break down the overall change into multiple components, thereby identifying the specific contribution of each. Based on the significant change periods of ASIR identified by joinpoint regression analysis, the changes in the number of incident cases in each period were decomposed into population age structure, population growth, and epidemiological trends, thereby objectively reflecting the specific drivers of change across different time periods.

APC analysis used an age‐by‐period table of incident counts and corresponding population exposures, comprising 18 5‐year age groups (5–9 to 90–94 years) and six 5‐year periods (1992–1996 to 2017–2021). The open‐ended 95+ group was excluded because a unique cohort midpoint could not be assigned. A log‐linear Poisson model was fitted to the age‐period‐specific incident counts, with the logarithm of the corresponding population exposure included as an offset to account for unequal population sizes. Age‐specific incidence rates were calculated within individual age strata and were used only to present the longitudinal age curve. They are distinct from ASIR, which is a weighted summary across age groups; therefore, ASIR was not used as the APC outcome. Model‐based standard errors and 95% confidence intervals were derived under the Poisson mean‐variance assumption and did not incorporate the full uncertainty of the modeled GBD estimates. The intrinsic estimator was used to address the linear dependency among age, period, and cohort. Accordingly, the estimated effects were interpreted as constrained descriptive patterns rather than causal effects. Analyses used the apc package in *R* 4.3.1 and the NCI APC tool (https://analysistools.cancer.gov/apc/).

Ultimately, the ASIRs of LBP in China from 2021 to 2036 were predicted using BAPC analysis, which was performed with the BAPC and INLA software packages in R. The model decomposes the population data into three main factors—age, period, and cohort effects—and generates predictions by analyzing their effects on LBP incidence. Population input data: Historical population data (1990–2021) were obtained from the GBD 2021 database. Future population projections (2022–2036) for China were extracted from the global population forecasts published by the IHME. The original age groups were aggregated into 20 age categories, consistent with the GBD incidence data (5‐year intervals, up to 95+ years), to construct a complete population matrix from 1990 to 2036. Age Standardization: ASIR was calculated using the GBD global standard population as weights, aggregated across the 20 age groups according to the formula $\text{ASIR}=\left(\sum_{\text{i}=1}^{20}\left({\text{r}}_{\text{i}}\times {\text{w}}_{\text{i}}\right)\right)/\left(\sum {\text{w}}_{\text{i}}\right)\times 100000,$ (*r*_*i*_: the incidence rate in the *i*th age group, *w*_*i*_: the population weight of the *i*th age group in the GBD standard population). This approach ensures comparability across different years and sexes. Posterior uncertainty from the Bayesian model was summarized using 95% credible intervals.

Except for the specifically identified software tools, all data processing and statistical analyses were conducted in RStudio using R. The primary datasets, analytical procedures, *R* scripts, and model settings are provided in the supporting materials accompanying this article. Because of file‐size limitations, further detailed data supporting the analyses are available from the first author upon reasonable request.

## 3. Results

From 1990 to 2021, the total number of individuals with LBP increased both globally and in China, reaching 628.84 million and 100.09 million, respectively, in 2021. This corresponded to a percentage increase of 62.60% globally and 46.59% in China over the study period. However, ASPR decreased both globally and in China, reaching 7463.13 and 5342.10 per 100,000 by 2021, representing decreases of 11.06% and 19.49%, respectively. The trend observed from 2019 to 2021 was consistent with that from 1990 to 2021.

**FIGURE 1:**
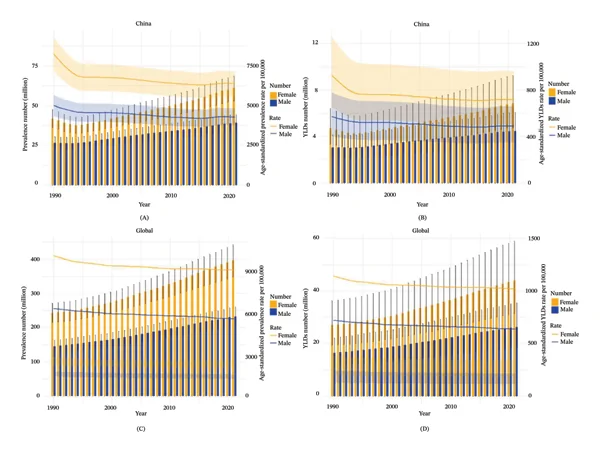
Trends in prevalence and YLDs due to LBP in China and the world, 1990–2021. LBP, low back pain; YLDs, years lived with disability.

Similarly, for YLDs with LBP, numbers increased globally and in China from 1990 to 2021, by 61.70% and 45.35%, respectively, reaching 70.15 million and 11.30 million in 2021. The ASYR declined by 11.22% globally and by 19.49% in China, with figures of 832.18 and 603.03 per 100,000 in 2021, respectively. Finally, the trend for 2019–2021 was the same as 1990–2021 (Table 2, Figure 1).

**TABLE 2:** Prevalence and YLDs due to low back pain in China and the world.

Region	Sex	Number of prevalent cases in 2021	Percentage change in prevalence number, 1990–2021 (%)	Percentage change in prevalence number, 2019–2021 (%)	Age‐standardized prevalence rate per 100,000 in 2021	Percentage change in age‐standardized prevalence rate, 1990–2021 (%)	Percentage change in age‐standardized prevalence rate, 2019–2021 (%)
China	Male	39,148,537 (33,850,361,44,326,367)	47.63 (41.31,55.15)	1.64 (0.10, 3.18)	4282.30 (3759.62,4838.55)	−14.48 (−16.26, −12.60)	−0.52 (−1.84, 0.72)
	Female	60,945,208 (52,941,431,68,505,911)	45.93 (38.41,53.53)	2.99 (1.64, 4.43)	6381.38 (5567.92,7153.22)	−22.62 (−24.45, −20.87)	−0.38 (−1.39, 0.73)
	Both	100,093,746 (87,128,173,113,014,316)	46.59 (40.01,53.85)	2.46 (1.25, 3.57)	5342.10 (4660.41,5976.28)	−19.49 (−21.04, −18.21)	−0.44 (−1.16, 0.40)

Global	Male	232,090,503 (203,351,350,259,931,953)	60.47 (57.38,63.95)	2.82 (2.37,3.29)	5640.23 (4965.63,6300.61)	−11.78 (−12.45, −11.12)	−0.40 (−0.82, 0.03)
	Female	396,747,972 (348,340,471,442,511,662)	63.88 (60.80,67.36)	3.14 (2.62,3.64)	9212.46 (8122.92,10,285.14)	−10.32 (−10.90, −9.76)	−0.24 (−0.67, 0.20)
	Both	628,838,475 (551,834,407,700,881,341)	62.60 (59.55,66.06)	3.02 (2.65,3.36)	7463.13 (6575.68,8321.80)	−11.06 (−11.58, −10.59)	−0.31 (−0.60, −0.02)

**Region**	**Sex**	**Number of YLDs in 2021**	**Percentage change in number of YLDs, 1990–2021 (%)**	**Percentage change in number of YLDs, 2019–2021 (%)**	**Age-standardized rate of YLDs per 100,000 in 2021**	**Percentage change in age-standardized rate of YLDs per 100,000, 1990–2021 (%)**	**Percentage change in age-standardized rate of YLDs per 100,000, 2019–2021 (%)**

China	Male	4,474,521 (3,156,562,6,067,442)	46.43 (39.97,53.99)	1.44 (−0.35, 3.19)	488.36 (346.52,657.58)	−14.58 (−16.59, −12.53)	−0.59 (−1.94, 0.84)
	Female	6,823,284 (4,775,816,9,218,929)	44.64 (37.34,52.72)	2.69 (1.27, 4.33)	716.15 (506.26,959.84)	−22.65 (−24.49, −20.69)	−0.51 (−1.59, 0.77)
	Both	11,297,805 (7,931,468,15,328,056)	45.35 (38.78,53.16)	2.19 (0.92, 3.49)	603.03 (427.63,810.16)	−19.49 (−21.09, −18.00)	−0.55 (−1.39, 0.43)

Global	Male	26,222,007 (18,702,285,35,396,348)	59.77 (56.51,63.65)	2.60 (2.03,3.15)	635.48 (453.91,854.29)	−11.81 (−12.59, −11.06)	−0.57 (−1.01, −0.08)
	Female	43,934,955 (31,447,685,58,945,143)	62.88 (59.62,66.80)	2.78 (2.22,3.31)	1021.52 (732.39,1370.45)	−10.57 (−11.22, −9.98)	−0.55 (−1.04, −0.08)
	Both	70,156,962 (50,194,205,94,104,688)	61.70 (58.59,65.45)	2.71 (2.27,3.08)	832.18 (595.85,1115.24)	−11.22 (−11.82, −10.66)	−0.56 (−0.94, −0.22)

*Note:* Data in parentheses are 95% uncertainty intervals. YLDs, years lived with disability.

Among the modifiable risk factors for LBP in the GBD 2021 study are occupational ergonomic factors, smoking, and high BMI. In 2021, the total number of YLDs attributable to all GBD‐estimated risk factors for LBP in China was 5.22 million (95% UI, 3.61–7.14). Of this total, occupational ergonomic factors accounted for 3.31 million YLDs (95% UI, 2.28–4.53), smoking for 1.85 million (95% UI, 1.08–2.70), and high BMI for 1.06 million (95% UI, 0.11–2.23). While the overall burden of YLDs attributable to all studied risk factors increased over the 1990–2021 period, trends diverged between 2019 and 2021; during this latter interval, YLDs attributable to occupational ergonomic factors declined by 0.61% (95% UI: −4.32 to 3.18), while all others increased. However, for ASYR, all risk factors declined from 1990 to 2021, except for the portion attributed to high BMI, which increased by 116.49% (95% UI, 78.16–143.81); this trend was consistent across both sexes. In addition, in 2019–2021, only the portion attributable to high BMI and the female portion of all factors increased from the previous year, while the others continued to decline (Table 3).

**TABLE 3:** YLDs and age‐standardized YLD rate for LBP and associated risk factors in China.

Sex	Numbers of YLDs, 2021	Percentage change in number, 1990–2021 (%)	Percentage change in number, 2019–2021 (%)	Age‐standardized rate per 100,000, 2021	Percentage change in age‐standardized rate, 1990–2021 (%)	Percentage change in age‐standardized rate, 2019–2021 (%)
*Low back pain attributable to all estimated risk factors*						
Male	2,600,000 (1,792,836,3,587,578)	31.79 (19.72,42.17)	1.06 (−1.26, 3.22)	270.01 (188.22,368.54)	−25.74 (−31.44, −20.67)	−0.71 (−2.68, 1.25)
Female	2,617,214 (1,772,924,3,683,494)	28.50 (4.89,52.40)	2.84 (−1.16, 6.33)	273.57 (188.31,377.67)	−28.99 (−41.64, −17.41)	0.54 (−3.00, 3.80)
Both	5,217,214 (3,613,107,7,139,183)	30.12 (13.71,44.86)	1.94 (−0.47, 4.45)	271.29 (188.79,371.86)	−27.31 (−35.54, −19.92)	−0.08 (−2.18, 1.86)

*Low back pain attributable to occupational ergonomic factors*						
Male	1,397,831 (934,966,1,938,798)	1.62 (−13.08,18.54)	−0.61 (−4.32,3.18)	149.83 (100.17,207.66)	−38.13 (−46.38, −28.54)	−1.36 (−4.78, 1.95)
Female	1,916,298 (1,268,797,2,697,981)	6.90 (−15.19,31.29)	0.99 (−3.78, 5.99)	208.00 (140.94,286.26)	−37.28 (−49.61, −24.13)	−0.39 (−5.01, 4.14)
Both	3,314,129 (2,275,896,4,532,168)	4.60 (−8.93,20.72)	0.31 (−2.96, 3.66)	178.44 (122.54,243.59)	−37.45 (−45.05, −29.25)	−0.83 (−3.81, 2.32)

*Low back pain attributable to smoking*						
Male	1,558,580 (931,831,2,265,680)	45.52 (36.55,53.96)	1.44 (−0.77, 3.43)	154.47 (92.95,224.17)	−26.11 (−29.50, −23.08)	−1.19 (−3.15, 0.55)
Female	294,550 (158,884,472,836)	30.81 (4.43,65.95)	1.71 (−3.20, 7.55)	27.02 (14.46,43.12)	−45.43 (−56.05, −31.89)	−3.22 (−7.39, 1.42)
Both	1,853,130 (1,084,581,2,703,583)	42.96 (33.75,52.38)	1.48 (−0.59, 3.43)	90.79 (53.26,131.01)	−30.69 (−34.26, −27.33)	−1.44 (−3.27, 0.20)

*Low back pain attributable to high body mass index*						
Male	358,719 (36840,745,322)	296.71 (232.92,349.23)	7.55 (4.23, 10.26)	37.45 (3.81,77.55)	118.68 (82.03,148.87)	5.94 (2.68, 8.57)
Female	703,298 (73131,1,478,285)	354.35 (273.07,417.05)	10.62 (7.77, 13.26)	67.51 (6.96,142.32)	113.64 (76.29,140.83)	6.88 (4.42, 9.22)
Both	1,062,018 (109,971,2,225,149)	333.10 (258.21,391.61)	9.57 (6.99, 11.71)	52.70 (5.41,110.10)	116.49 (78.16,143.81)	6.57 (4.11, 8.55)

*Note:* Data in parentheses are 95% uncertainty intervals. YLDs, years lived with disability.

**FIGURE 2:**
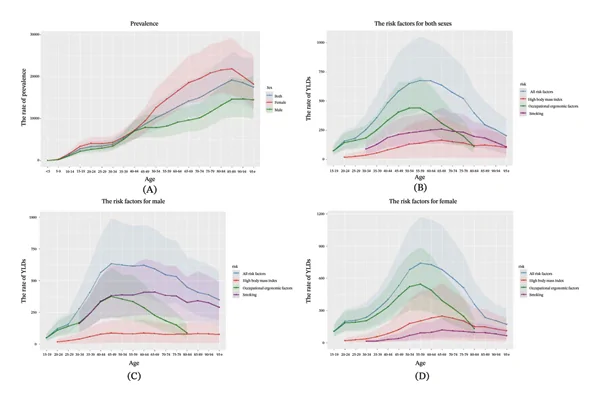
Longitudinal age curves for prevalence and YLD rate of low back pain attributed to different risk factors in China. YLDs, years lived with disability.

With 5‐year age groups, Figure 2 illustrates trends in LBP prevalence by gender and in YLD rates attributable to different risk factors across age groups in China in 2021. As shown in Figure 2A, LBP was observed in the 10–14 years age group, with the prevalence rate increasing more rapidly from the 35–39 years age group onward and reaching its highest point in the 85–89 years age group; this pattern was consistent for both sexes. Regarding gender differences, little disparity in prevalence rates between men and women was observed until age 40–44 years, after which the prevalence rate was higher in women than in men.

Figures 2B–D display the trends in YLD rates attributed to different modifiable risk factors by age and gender, respectively. Among cases of LBP attributed to occupational ergonomic factors, the affected population spanned from the 15–19 age group to the 80–84 age group. LBP attributed to high BMI was first observed in the 20–24 age group, and that attributed to smoking was first documented in the 30–34 age group, with cases of both still present at 95+ years. Overall (both sexes), the peak YLD rate across all risk factors was observed in the 55–64 age range. The YLD rate attributed to occupational ergonomic factors was higher than that for high BMI and smoking until age 70–75, and after age 75–79, smoking surpassed occupational ergonomic factors as the highest. In men, smoking emerged as the main risk factor after the age of 45–49 years, while high BMI was lower in all age groups. In women, occupational ergonomic factors were the main risk factors by age 75–79 years, and the most significant difference from men was smoking, with women’s YLD rate attributable to smoking at the lowest levels in all age groups compared to other risk factors.

**FIGURE 3:**
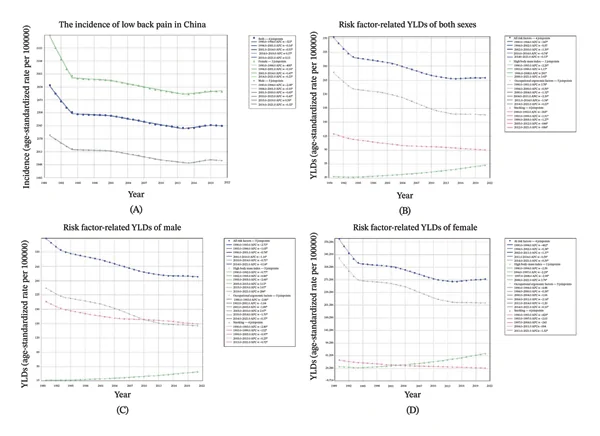
Joinpoint regression analysis of age‐standardized incidence rates by sex and age‐standardized YLD rates attributed to different risk factors for LBP in China. YLDs, years lived with disability; LBP, low back pain; APC, annual percentage change. ^∗^Statistical significance regarding the observed differences (*p* < 0.05).

The results of the joinpoint regression analysis of ASIR by sex and ASYR attributable to various risk factors for LBP in China from 1990 to 2021 are shown in Figure 3. A significant decrease in both male and female ASIR was observed from 1990–1994 (both sexes: APC = −3.23; female: APC = −4.00; male: APC = −2.08); the overall decreasing trend slowed from 1994–2014; however, there was an increasing trend from 2014 onwards (both sexes: APC = 0.37). Notably, the GBD 2021 data indicated a slight overall decline from 2019 to 2021; however, this decline was observed primarily among males, with no significant change among females.

The ASIR among females increased from 2014 (2014–2021: APC = 0.23). Among males, after an upward trend from 2015 to 2019 (APC = 0.59), a subsequent decline was observed from 2019 to 2021 (APC = −0.32); however, the magnitude of all changes was small.

For modifiable risk factors, the ASYR attributable to all risk factors for both sexes declined significantly from 1990 to 1994 (APC = −3.47), with a slower downward trend from 1994 to 2014 and a small increase from 2014 to 2021 (APC = 0.13). However, the ASYR attributable to high BMI exhibited a brief decline in both males and females (both sexes: APC = −2.26 (1990–1993); male: APC = −0.77 (1990–1992); female: APC = −2.59 (1990–1994)), before resuming an upward trajectory. In males, both occupational ergonomic factors and smoking were at higher levels compared to high BMI. However, the levels of occupational ergonomic factors were significantly higher than those of smoking and high BMI in females.

**FIGURE 4:**
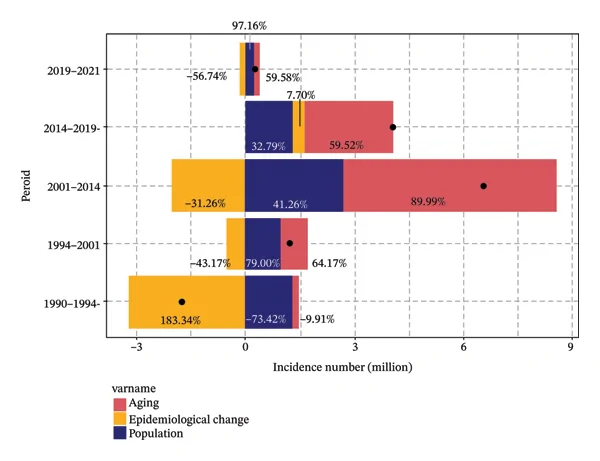
Decomposition analysis for incident cases of low back pain in different periods in China. ±  indicates trend of change over the corresponding time period. • indicates the total number of incident cases.

Based on the joinpoint regression analysis, we identified four significant turning points in ASIR (1994, 2001, 2014, and 2019), which defined five periods for the decomposition analysis of incident case counts (1990–1994, 1994–2001, 2001–2014, 2014–2019, and 2019–2021) (Figure 4, Table 4). The results of the analysis showed that over the past 30 years, both population growth and aging in China have had a positive effect on the increase in LBP incident cases. Despite the overall downward trend in ASIR, incident cases declined only during 1990–1994, then increased from 1994–2021. There was a significant decline in ASIR from 1990–1994, with epidemiological changes (including factors other than population and aging) predominating, accounting for 183.34% of the total decline, while the effects of population and aging accounted for −73.42% and −9.91%, respectively. Their positive impact on incident case growth dominated after 1994 as aging and population growth intensified, resulting in an overall upward trend. The impact of aging increased annually from 1990 to 2014, peaking at 89.99% in 2001–2014. In contrast, population growth exerted the greatest impact on incident cases during 2019–2021, accounting for 97.16% of the total change.

**TABLE 4:** Changes in incident cases according to population‐level determinants and causes.

Period	Overall change in incident cases	Change due to population‐level determinants (% contribution to the total changes)		
		Aging	Population	Epidemiological change
1990–1994	−1753,132.52	173,796.15 (−9.91)	1,287,220.89 (−73.42)	−3214,149.56 (183.34)
1994–2001	1,201,488.09	770,946.80 (64.17)	949,172.72 (79.00)	−518631.43 (−43.17)
2001–2014	6,552,283.59	5,896,705.59 (89.99)	2,703,502.08 (41.26)	−2047924.08 (−31.26)
2014–2019	4,059,484.97	2,416,113.06 (59.52)	1,330,905.65 (32.79)	312,466.26 (7.70)
2019–2021	255,321.81	152,112.05 (59.58)	248,073.02 (97.16)	−144863.26 (−56.74)

**FIGURE 5:**
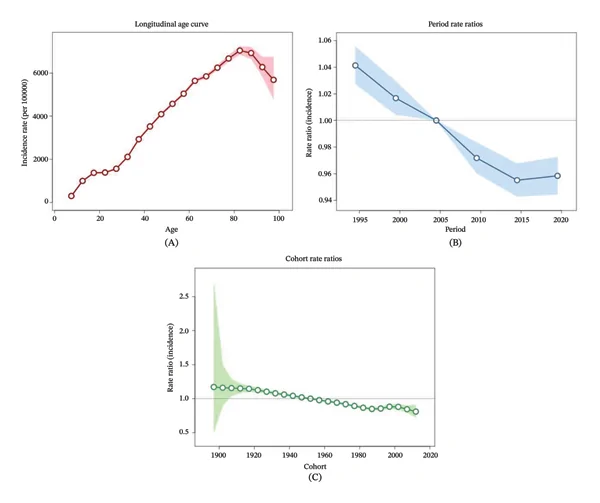
Age‐specific incidence rate and period and cohort rate ratios for LBP from the age‐period‐cohort analysis. LBP, low back pain. Note: Period and cohort effects are expressed as rate ratios (RRs), not age‐standardized rates.

Figure 5 shows the age‐specific incidence rate (Figure 5A) and period and cohort rate ratios (Figure 5B,C) from the APC analysis. The APC estimates were obtained from incident counts with population exposure as an offset; period and cohort effects are therefore reported as rate ratios rather than age‐standardized rates. As shown in Figure 5A, the estimated age‐specific incidence rate of LBP increased from 5 to 9 years of age (incidence rate = 297.63 per 100,000), peaked at 80–84 years of age (incidence rate = 7040.94 per 100,000), and then declined.

The period effect analysis of rate ratios (Figure 5B) demonstrated a nonlinear trend, shifting from a downward to an upward trajectory. Taking the period rate ratio for 2002–2006 as 1, its lowest point occurred during 2012–2016, with an RR of 0.96 (95% CI: 0.94–0.97).

Analysis of birth cohort effects across age groups (Figure 5C) showed an overall decreasing trend in the cohort rate ratio, indicating higher relative incidence in earlier birth cohorts than in later birth cohorts. With the 1952 birth cohort established as the reference group (RR = 1), the earliest birth cohort born in 1897–1901 exhibited the highest associated risk, at an RR of 1.17 (95% CI: 0.506–2.71). Conversely, the lowest risk was observed in the 2012–2016 birth cohort, with an RR of 0.81 (95% CI: 0.72–0.91). Detailed results of the APC model, including age‐specific incidence rates, period and cohort rate ratios with 95% confidence intervals, intrinsic estimator coefficients, and Wald tests for model components, are provided in Supporting Tables S1–S5.

**FIGURE 6:**
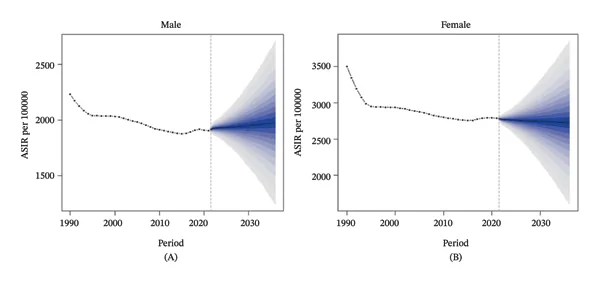
Trends in ASIR from 2021 to 2036 among males and females, predicted by Bayesian age‐period‐cohort (BAPC) models. The shaded bands represent posterior uncertainty, which widens with the projection horizon. ASIR: age‐standardized incidence rate.

Finally, the BAPC model was used to project ASIR from 2021 to 2036 (Figure 6). The posterior mean pattern for men showed a slight increase from 1906.32 per 100,000 in 2021 (95% credible interval: 1905.52–1907.12) to 1975.70 per 100,000 in 2036 (95% credible interval: 1359.80–2591.79). For women, the posterior mean pattern showed a slight decrease from 2786.47 per 100,000 in 2021 (95% credible interval: 2785.51–2787.43) to 2725.88 per 100,000 in 2036 (95% credible interval: 1773.58–3677.57). Nevertheless, uncertainty widened markedly over the projection period, especially in the sex‐specific estimates by 2036. The broad and overlapping credible intervals are compatible with a range of trajectories and do not support strong conclusions about sustained increases in men or decreases in women. These forecasts should therefore be interpreted as uncertain scenario projections rather than definitive directional trends. Detailed data are provided in Supporting Table S6.

## 4. Discussion

In recent years, several GBD‐based studies have analyzed the disease burden of LBP in China. In this respect, Zhang et al. used GBD 2019 data to explore temporal trends in LBP‐related YLDs and attributable risk factors in China from 1990 to 2019, while Wei et al. focused on the incidence trends and projections of spinal pain (including neck and LBP) [8, 14]. Additionally, the GBD 2021 Low Back Pain Collaborator Group provided comprehensive global and regional burden data through 2020 [3].

Building on these previous studies, the present analysis provides a complementary assessment of LBP burden in China using GBD 2021 estimates. Rather than introducing a new modeling framework, this study integrates several established epidemiological approaches to further characterize temporal changes and potential drivers of LBP burden. Specifically, compared with previous China‐focused GBD analyses, we additionally examined the contribution of population aging, population growth, and epidemiological changes to variations in incident cases through decomposition analysis. Furthermore, APC analysis was applied to explore long‐term changes in incidence patterns across different generations, and risk‐factor‐related YLDs were evaluated by sex and age group to provide more detailed information for targeted prevention strategies.

### 4.1. Trends in the Burden of LBP Over Time in China

Our study found that the ASPR of LBP in China is comparable to the global average in 2021. Over the past 3 decades, the ASPR, ASIR, and ASYR of LBP in China generally declined. The timing of these trends partly coincided with the implementation of major national health reforms and chronic disease prevention initiatives, raising the hypothesis that such policies may have contributed to the observed population‐level changes. However, because this is a descriptive ecological analysis, temporal alignment alone cannot establish a causal relationship or demonstrate policy effectiveness, and concurrent demographic, socioeconomic, occupational, and healthcare‐related changes may also have influenced these trends. The pace of decline has slowed in recent years, while prevalent cases and YLDs have continued to increase (Table 2, Figure 1), indicating that LBP remains a substantial public health burden in China.

The joinpoint regression analysis identified four significant ASIR turning points in China’s total population, which defined five periods over the last 30 years. Notably, the fourth inflection point occurred in 2019; the GBD 2021 data showed that this shifted the overall trend from increasing to decreasing over 2019–2021, albeit by a small margin (Figure 3).

Decomposition analysis across the five periods defined by the four ASIR turning points revealed that the large decline in incidence rate from 1990 to 1994 was mainly attributed to epidemiological changes, and the total number of incident cases also decreased. Wei et al. reported that China’s production and lifestyles changed dramatically during this period, and the degree of automation and mechanization in industrial and agricultural production increased significantly, greatly reducing the labor intensity of farmers and workers. Notably, China’s health care remained relatively underdeveloped during this period, while population aging was not yet pronounced. These concurrent socioeconomic and occupational changes may have been associated with the rapid decline in the ASIR of LBP in China from 1990 to 1994, although their independent contribution cannot be determined from this ecological analysis [14].

The decline in ASIR for LBP slowed from 1994 to 2014, and decomposition analysis showed an increasing impact of population aging, with this factor accounting for 89.99% of the decline from 2001 to 2014, while the total number of incident cases continued to increase. James P. Smith noted in his study on population aging in China that improvements in medical care extended life expectancy, while the family planning policy reduced birth rates. These two factors may have contributed to the acceleration of population aging in China around the year 2000 [15], although their independent effects cannot be quantified in the present analysis.

A 2016 editorial in The Lancet [6] asserted that mitigating the challenges of China’s aging population should not rely primarily on the family, but instead requires urgent social and political transformation to adapt to rapid demographic changes. In response to these demographic pressures, the Chinese government has implemented several policies and plans to address them. China’s Chronic Disease Prevention and Control Work Plan (2012–2015), which was implemented in 2012, included the prevention and treatment of chronic noncommunicable diseases (including LBP) as an important part of the national health work, proposing to strengthen the early detection, standardized treatment and management of chronic diseases [16]. The “Healthy China 2030” planning outline proposed in 2016 emphasizes the importance of promoting health for all and proposes a number of measures, including strengthening chronic disease management, aiming to improve the health of the whole population and reduce the burden of chronic diseases, including the prevention and treatment of common diseases such as LBP [7]. The Intermediate‐ and Long‐Term Plan for the Prevention and Treatment of Chronic Diseases in China (2017–2025), which was implemented in 2017, clearly defines the goals, tasks, and measures for the prevention and treatment of chronic diseases, and emphasizes key aspects such as health education, lifestyle intervention, early diagnosis and treatment, standardized diagnosis and treatment, and rehabilitation management, which are of great significance for the prevention and control of chronic diseases such as LBP [17]. Although the implementation periods of these policies overlap with parts of the observed decline in age‐standardized rates, this temporal correspondence is hypothesis‐generating only and should not be interpreted as evidence that the policies caused the decline or were effective.

In recent years, the contribution of population aging to changes in LBP incident cases has weakened during a period in which several “healthy aging” initiatives were introduced. This temporal overlap is consistent with, but does not demonstrate, a possible contribution from these initiatives; other concurrent demographic, socioeconomic, and healthcare changes may also have played a role. GBD 2021 data also indicated that population growth became a major contributor to the change in LBP incident cases during 2019–2021 (Figure 4). Future planning should therefore account for demographic expansion when allocating prevention, treatment, and rehabilitation resources.

### 4.2. Risk Factors of LBP Burden

The results of the present study showed that occupational ergonomic factors (OEFs) remained the main risk factor for overall LBP in China until age 70–75 years (Figure 2B). However, the joinpoint regression analysis shows that the ASYR attributable to OEFs has been decreasing since 1990. It has been shown that the decrease in OEFs over time primarily reflects a decrease in the proportion of employment in high‐risk occupations (e.g., agriculture and labor‐intensive work) [10]. This finding is closely related to the changes in labor production methods in China. The proportion of agricultural and manual labor jobs in China decreased from 57% in 1990 to 32% in 2015 [18], which may account for the overall decrease in the incidence rate of LBP in China. This trend is also reflected in the birth cohort curves shown in Figure 5C.

However, an estimated 34 million workers were laid off by the Chinese government between 1995 and 2001 in an effort to streamline the workforce of state‐owned enterprises [19], which forced many people with comfortable jobs into manual labor. Together with the slowing down of the decline in the number of people engaged in agricultural production in China after 1994 [8], these labor‐market changes may have been associated with the slower decline in OEFs from 1994–2002, during which the APC changed from −3.78 to −0.59 (Figure 3B). It should be noted that this temporal association does not establish causality. Second, with advances in information technology, computerized office work has become the primary mode of work for urban office workers. The proportion of the population who spend a significant amount of time sitting at a computer at work each day has been increasing, leading to an escalation in the burden associated with LBP [14]. This finding may account for the decline in ASYR, which has continued to slow down since 2011.

It is now well established that smoking is associated with LBP [20]. The sustained decline observed in both men and women occurred during a period of relevant public awareness campaigns (Figure 3B–D), raising the hypothesis that these initiatives may have contributed, although temporal concurrence alone does not demonstrate their effectiveness. According to the 2021 data, smoking remained a major risk factor for LBP in men. Notably, among men over 50 years of age, the ASYR attributable to smoking was higher than that of high BMI and OEFs. This finding highlights smoking as a potentially important target for further etiological and prevention research in men. However, the GBD‐attributed burden should not be interpreted as the amount of burden that would be directly reversible through smoking‐control interventions.

In addition, high BMI was the only one of the three LBP‐related risk factors to show a year‐over‐year increase (Table 3, Figure 3B–D). These changes may be related to concurrent shifts in China’s production patterns and lifestyles, although the present analysis cannot establish their causal contribution. Although the declining proportion of physical work may reduce OEF‐related burden, it may also reduce routine physical activity and, together with dietary changes, contribute to rising obesity in China. Sex‐specific differences in physiology and lifestyle habits may contribute to a higher burden of YLDs attributable to high BMI in females than in males (Figures 2C,D and 3C,D).

Notably, the GBD framework includes only a subset of modifiable risk factors for which sufficient data are available for systematic estimation. Thus, the present analysis neither accounts for all potential contributors to LBP burden, nor establishes a comprehensive causal framework. The attribution estimates are subject to assumptions regarding the exposure distribution, the constancy of relative risk, and the independence of risk factors. Accordingly, population‐attributable fractions represent model‐based counterfactual estimates and should not be interpreted as directly reversible causal effects or as a stand‐alone ranking of intervention priorities. Nevertheless, they provide a consistent basis for identifying population‐level patterns among the quantifiable risks included in GBD and for informing further research and context‐specific prevention planning.

Finally, a heavier burden of LBP was observed in women than in men. Sex differences may stem from differences in physiologic characteristics, disease exposure risk, and health management between men and women [21]. Specifically, lower lumbar muscle mass, menstrual cycle changes, and specific hormones may play important roles in the etiology and pathophysiology of LBP in women. In addition, women’s greater sensitivity to pain and greater willingness to report painful symptoms may also contribute [14]. In addition, the prevalence of osteoporosis and lumbar microfractures has been reported to be significantly higher in older women [22]. However, the BAPC posterior means suggested a small increase in males and a small decrease in females from 2021 to 2036, but the credible intervals widened substantially and overlapped broad ranges of possible values. These sex‐specific projections suggest potential trends that may inform surveillance but should be interpreted cautiously given the widening credible intervals. Therefore, sex‐specific strategies for LBP prevention and control should also be a focus of public health interventions.

Overall, this study characterizes temporal trends in disease burden and associated factors rather than estimating causal policy effects. Associations between national policies and observed changes are presented as hypothesis‐generating interpretations based on temporal correspondence and existing literature. Such alignment may suggest a possible contribution from policy initiatives, but it cannot demonstrate effectiveness or separate policy effects from concurrent demographic, socioeconomic, occupational, and healthcare‐related changes.

### 4.3. Limitations

Some limitations of this study should be acknowledged. First, obtaining complete raw data for each region is challenging; therefore, estimates of the LBP burden from the GBD database were based on modeled data rather than entirely on observed data [23]. However, by using statistical adjustments for key covariates and harmonizing between‐study heterogeneity through a formally defined reference definition, the modeled data provide the most accurate estimates of global prevalence and burden of LBP [3]. GBD estimates for occupational ergonomic factors, smoking, and high BMI rely on complex modeling approaches that integrate multisource data with varying quality across different periods. The assumptions these models make about exposure distributions, relative risks, and counterfactual scenarios may not fully capture the complex relationships among these factors and LBP in China, nor adequately account for interactions between risk factors and unmeasured confounders. Second, with the rapid development of China’s economy and medical care in the past 30 years, people’s opportunities and awareness of access to medical care for LBP have changed considerably, leading to Berkson’s bias in the data at different periods. Third, our study was conducted at the national level, and given the uneven economic development across regions in China, the results are not representative of each region. For the prevention and treatment of LBP across China, it still needs to be addressed specifically according to regional conditions. Fourth, the risk factor attribution in GBD relies on modeled exposure data and fixed relative risks, which may not fully capture time‐varying effects, measurement error across data sources, or potential interactions among risk factors (e.g., between high BMI and occupational ergonomic exposures). These factors may introduce bias in the estimated attributable burden and limit the precision of cross‐factor comparisons. Fifth, because the APC inputs were modeled GBD estimates, the Poisson confidence intervals do not incorporate the GBD UIs, potential extra‐Poisson variation, or correlations across age‐period cells. The IE estimates also depend on the identification constraint and should be interpreted as descriptive rather than causal. Moreover, the national‐level ecological design precludes individual‐level or policy‐specific causal inference. Temporal correspondence between policy implementation and changes in LBP rates is therefore hypothesis‐generating only and should not be interpreted as evidence of policy effectiveness.

## 5. Conclusion

In conclusion, the absolute burden of LBP in China has continued to rise over the past 30 years, while age‐standardized rates have generally declined. The downward trends partly coincided temporally with several national health and chronic disease policies, raising the hypothesis that related initiatives may have contributed to the observed population‐level changes. However, this temporal correspondence is not evidence of effectiveness, and causal policy effects cannot be determined from the present ecological analysis. The contributions of OEFs and population aging have declined in recent periods, whereas those of high BMI and population growth have increased, and smoking‐attributable burden remains higher in men than in women. To address the continuing burden, occupational health, weight management, and tobacco control remain important areas for prevention, while recognizing that GBD includes only a limited set of quantifiable risk factors.

**Nomenclature**
LBP: Low back pain
GBD: Global Burden of Disease
BAPC: Bayesian age‐period‐cohort
YLDs: Years lived with disability
ASPR: Age‐standardized prevalence rate
ASYR: Age‐standardized YLD rate
ASIR: Age‐standardized incidence rate
APC: Annual percentage change
CPC: Communist Party of China
OEFs: Occupational ergonomic factors

## Author Contributions

Antao Lin made the primary contribution to this study, including data organization and analysis, and drafted the main manuscript. Yuanye Ma and Chengwei Zhang provided theoretical guidance. Wenke Yang contributed to data collection. Shuo Han and Hao Zhang contributed to data analysis. Zhiming Liu and Shuai Tang contributed to data interpretation. Wenhua Gong contributed to manuscript review and editing. Xuexiao Ma supervised the study and critically revised the manuscript.

## Funding

This work was supported by grants from the Taishan Scholars Program of Shandong Province (No. tstp 20230664) and Qingdao Science and Technology Benefit the People Demonstration Project (No. 23‐2‐8‐smjk‐7‐nsh).

## Disclosure

All authors reviewed and approved the final manuscript.

## Ethics Statement

Data related to the burden of low back pain used in this study were obtained from the Global Burden of Disease 2021 (GBD 2021) database, and no new ethical approvals were required as the study used publicly available data. This study conformed to the principles of the Declaration of Helsinki. Patient consent for publication was not required.

## Consent

Please see the Ethics Statement.

## Conflicts of Interest

The authors declare no conflicts of interest.

## Supporting Information

Additional supporting information can be found online in the Supporting Information section.

## Supporting information


**Supporting Information** Table S1. Detailed data related to the longitudinal age curve. Table S2. Detailed data related to period rate ratios. Table S3. Detailed data related to cohort rate ratios. Table S4. Intrinsic estimator (IE) coefficients for age, period, and cohort effects from the APC model. Table S5. Wald tests for APC model components. Table S6. Detailed data related to trends of ASIR per 100,000 from 2021 to 2036 predicted by Bayesian age‐period‐cohort (BAPC) models.

## Data Availability

The GBD 2021 estimates analyzed in this study are publicly available from the Institute for Health Metrics and Evaluation (IHME) through the Global Health Data Exchange (GHDx) GBD 2021 data portal (https://ghdx.healthdata.org/gbd-2021) and the GBD Results Tool (https://vizhub.healthdata.org/gbd-results/). The primary datasets, *R* scripts, and model settings are provided in the supporting materials. Owing to file‐size limitations, additional detailed data supporting the analyses are available from the first author upon reasonable request. No new individual‐level data were generated in this study.
