# Supplementary material for: The Burden, Risk Factor Analysis, and Prediction of Low Back Pain in China From 1990 to 2021
Source: Pain Res Manag. 2026 Sep 16;2026:6863134. doi: 10.1155/prm/6863134 (PMC13580342; doi:10.1155/prm/6863134)
Supplement: Supplementary file 1 [file PRM-2026-6863134-s001.zip › Supplementary material.docx]

Table S1. Detailed data related to longitudinal age curve.

| Age | Rate | 95%CILo | 95%CIHi |
| --- | --- | --- | --- |
| 7.5 | 297.6294 | 282.5861 | 313.4735 |
| 12.5 | 995.6835 | 964.039 | 1028.3667 |
| 17.5 | 1372.8912 | 1335.4678 | 1411.3634 |
| 22.5 | 1385.2801 | 1350.532 | 1420.9222 |
| 27.5 | 1561.9935 | 1526.9113 | 1597.8818 |
| 32.5 | 2111.2082 | 2069.4137 | 2153.8468 |
| 37.5 | 2930.6895 | 2878.9009 | 2983.4097 |
| 42.5 | 3520.9298 | 3466.8067 | 3575.898 |
| 47.5 | 4091.8537 | 4032.7958 | 4151.7765 |
| 52.5 | 4570.6784 | 4506.7713 | 4635.4917 |
| 57.5 | 5040.488 | 4969.8278 | 5112.153 |
| 62.5 | 5636.5495 | 5555.8857 | 5718.3844 |
| 67.5 | 5843.9562 | 5753.5218 | 5935.812 |
| 72.5 | 6246.9488 | 6127.5367 | 6368.6881 |
| 77.5 | 6671.38 | 6525.0234 | 6821.0193 |
| 82.5 | 7040.9348 | 6852.2886 | 7234.7746 |
| 87.5 | 6925.6367 | 6661.0335 | 7200.7509 |
| 92.5 | 6274.8235 | 5840.1907 | 6741.8022 |
| 97.5 | 5682.4052 | 4787.5843 | 6744.4722 |

Table S2. Detailed data related to Period Rate Ratios.

| Period | Rate Ratio | 95%CILo | 95%CIHi |
| --- | --- | --- | --- |
| 1994.5 | 1.0413 | 1.0274 | 1.0555 |
| 1999.5 | 1.0168 | 1.0044 | 1.0293 |
| 2004.5 | 1 | 1 | 1 |
| 2009.5 | 0.9718 | 0.9603 | 0.9833 |
| 2014.5 | 0.9551 | 0.9429 | 0.9676 |
| 2019.5 | 0.9584 | 0.9445 | 0.9725 |

Table S3. Detailed data related to Cohort Rate Ratios.

| Cohort | Rate Ratio | 95%CILo | 95%CIHi |
| --- | --- | --- | --- |
| 1897 | 1.1711 | 0.506 | 2.7102 |
| 1902 | 1.1604 | 0.8969 | 1.5013 |
| 1907 | 1.1553 | 1.0381 | 1.2858 |
| 1912 | 1.1502 | 1.0847 | 1.2198 |
| 1917 | 1.1442 | 1.1002 | 1.19 |
| 1922 | 1.1253 | 1.0923 | 1.1594 |
| 1927 | 1.1023 | 1.0761 | 1.1292 |
| 1932 | 1.0786 | 1.0567 | 1.1009 |
| 1937 | 1.0599 | 1.0406 | 1.0796 |
| 1942 | 1.0412 | 1.0236 | 1.0591 |
| 1947 | 1.0185 | 1.0028 | 1.0344 |
| 1952 | 1 | 1 | 1 |
| 1957 | 0.9784 | 0.964 | 0.9931 |
| 1962 | 0.9599 | 0.9452 | 0.9749 |
| 1967 | 0.9415 | 0.9265 | 0.9567 |
| 1972 | 0.9186 | 0.9023 | 0.9351 |
| 1977 | 0.893 | 0.8747 | 0.9117 |
| 1982 | 0.8671 | 0.8469 | 0.8877 |
| 1987 | 0.8496 | 0.8282 | 0.8715 |
| 1992 | 0.8556 | 0.8303 | 0.8816 |
| 1997 | 0.8826 | 0.8509 | 0.9154 |
| 2002 | 0.88 | 0.8415 | 0.9203 |
| 2007 | 0.8449 | 0.7963 | 0.8964 |
| 2012 | 0.8107 | 0.7226 | 0.9095 |

Table S4. Intrinsic estimator (IE) coefficients for age, period, and cohort effects from APC model

|  | Parameter | SD | 95% CI Lo | 95% CI Hi |
| --- | --- | --- | --- | --- |
| Intercept | -3.441 | 0.007 | -3.454 | -3.428 |
| LAT | 0.027 | 0 | 0.026 | 0.028 |
| NetDrift | -0.004 | 0 | -0.004 | -0.003 |
| CAT | 0.031 | 0 | 0.03 | 0.031 |

Table S5. Wald tests for APC model components

|  | X2 | df | P-Value |
| --- | --- | --- | --- |
| NetDrift = 0 | 93.2182 | 1 | <0.001 |
| All Age Deviations = 0 | 6558.3662 | 17 | <0.001 |
| All Period Deviations = 0 | 23.3391 | 4 | <0.001 |
| All Cohort Deviations = 0 | 44.6657 | 22 | 0.0029 |
| All Period RR = 1 | 113.6236 | 5 | <0.001 |
| All Cohort RR = 1 | 414.9839 | 23 | <0.001 |
| All Local Drifts = Net Drift | 44.3338 | 19 | 0.0008 |

Table S6. Detailed data related to Trends of ASIR per 100000 from 2021 to 2036 predicted by Bayesian age-period-cohort (BAPC) models.

| Year | Male | | | |  | Female | | | |
| --- | --- | --- | --- | --- | --- | --- | --- | --- | --- |
|  | mean | sd | 95%CILo | 95%CIHi |  | mean | sd | 95%CILo | 95%CIHi |
| 1990 | 2230.74 | 0.74 | 2229.521187 | 2231.968448 |  | 3503.97 | 0.87 | 3502.541407 | 3505.395729 |
| 1991 | 2176.03 | 0.72 | 2174.847108 | 2177.217252 |  | 3343.21 | 0.84 | 3341.831861 | 3344.585229 |
| 1992 | 2125.96 | 0.7 | 2124.804466 | 2127.108159 |  | 3195.82 | 0.81 | 3194.485245 | 3197.14746 |
| 1993 | 2084.34 | 0.68 | 2083.212392 | 2085.457876 |  | 3073.71 | 0.79 | 3072.420888 | 3075.004263 |
| 1994 | 2054.93 | 0.67 | 2053.829998 | 2056.025387 |  | 2988.7 | 0.77 | 2987.442142 | 2989.960728 |
| 1995 | 2041.6 | 0.66 | 2040.519013 | 2042.674624 |  | 2952.58 | 0.75 | 2951.342195 | 2953.818522 |
| 1996 | 2038.54 | 0.64 | 2037.480036 | 2039.601794 |  | 2946.6 | 0.74 | 2945.377224 | 2947.82124 |
| 1997 | 2036.92 | 0.64 | 2035.875174 | 2037.964965 |  | 2943.31 | 0.73 | 2942.0991 | 2944.511726 |
| 1998 | 2035.91 | 0.63 | 2034.883277 | 2036.943217 |  | 2941.32 | 0.72 | 2940.126165 | 2942.508467 |
| 1999 | 2034.67 | 0.62 | 2033.658367 | 2035.689523 |  | 2939.32 | 0.71 | 2938.140474 | 2940.491337 |
| 2000 | 2032.43 | 0.61 | 2031.431075 | 2033.436629 |  | 2936.08 | 0.71 | 2934.915025 | 2937.238065 |
| 2001 | 2026.71 | 0.6 | 2025.720753 | 2027.700831 |  | 2928.64 | 0.7 | 2927.491107 | 2929.78386 |
| 2002 | 2016.88 | 0.59 | 2015.90871 | 2017.861356 |  | 2916.53 | 0.69 | 2915.400752 | 2917.66171 |
| 2003 | 2004.88 | 0.59 | 2003.91656 | 2005.842399 |  | 2902.02 | 0.68 | 2900.908473 | 2903.137836 |
| 2004 | 1992.53 | 0.58 | 1991.575592 | 1993.476337 |  | 2887.38 | 0.67 | 2886.283127 | 2888.481257 |
| 2005 | 1981.62 | 0.57 | 1980.678834 | 1982.55649 |  | 2874.97 | 0.66 | 2873.880011 | 2876.05202 |
| 2006 | 1969.72 | 0.56 | 1968.794 | 1970.644359 |  | 2861.67 | 0.65 | 2860.597563 | 2862.741392 |
| 2007 | 1954.62 | 0.55 | 1953.709647 | 1955.528321 |  | 2844.78 | 0.64 | 2843.722845 | 2845.836381 |
| 2008 | 1938.65 | 0.54 | 1937.760807 | 1939.54744 |  | 2827.02 | 0.63 | 2825.976796 | 2828.060801 |
| 2009 | 1924.18 | 0.53 | 1923.30621 | 1925.062469 |  | 2811.05 | 0.63 | 2810.019763 | 2812.076186 |
| 2010 | 1913.61 | 0.53 | 1912.745978 | 1914.476368 |  | 2799.71 | 0.62 | 2798.690095 | 2800.725309 |
| 2011 | 1905.5 | 0.52 | 1904.646979 | 1906.353371 |  | 2790.24 | 0.61 | 2789.229872 | 2791.245056 |
| 2012 | 1897.17 | 0.51 | 1896.331288 | 1898.014428 |  | 2779.29 | 0.61 | 2778.293004 | 2780.287821 |
| 2013 | 1889.19 | 0.51 | 1888.35844 | 1890.01984 |  | 2768.6 | 0.6 | 2767.612805 | 2769.587762 |
| 2014 | 1882.14 | 0.5 | 1881.317486 | 1882.959467 |  | 2759.88 | 0.59 | 2758.905984 | 2760.863007 |
| 2015 | 1876.63 | 0.49 | 1875.817771 | 1877.444155 |  | 2754.95 | 0.59 | 2753.980399 | 2755.924517 |
| 2016 | 1880.76 | 0.49 | 1879.948725 | 1881.566868 |  | 2759.74 | 0.59 | 2758.773861 | 2760.709948 |
| 2017 | 1895.58 | 0.49 | 1894.772823 | 1896.391398 |  | 2773.5 | 0.59 | 2772.536254 | 2774.469099 |
| 2018 | 1910.98 | 0.49 | 1910.16502 | 1911.786034 |  | 2788.04 | 0.59 | 2787.070174 | 2789.000793 |
| 2019 | 1916.82 | 0.49 | 1916.009965 | 1917.627752 |  | 2795.1 | 0.59 | 2794.14163 | 2796.067565 |
| 2020 | 1911.21 | 0.49 | 1910.401373 | 1912.010179 |  | 2791.4 | 0.58 | 2790.435971 | 2792.354189 |
| 2021 | 1906.32 | 0.49 | 1905.524085 | 1907.124557 |  | 2786.47 | 0.58 | 2785.514595 | 2787.425964 |
| 2022 | 1925.84 | 28.24 | 1879.460791 | 1972.236218 |  | 2771.77 | 35.28 | 2713.625639 | 2829.852656 |
| 2023 | 1928.5 | 39.65 | 1863.398075 | 1993.633242 |  | 2768.21 | 54.22 | 2678.765513 | 2857.548806 |
| 2024 | 1931.24 | 54.53 | 1841.697322 | 2020.81655 |  | 2764.74 | 78.46 | 2635.257683 | 2894.07931 |
| 2025 | 1934.14 | 72.06 | 1815.820819 | 2052.508096 |  | 2761.42 | 106.62 | 2585.41708 | 2937.23189 |
| 2026 | 1937.27 | 91.77 | 1786.58501 | 2088.014375 |  | 2758.06 | 138.01 | 2530.194976 | 2985.693062 |
| 2027 | 1940.62 | 113.41 | 1754.418227 | 2126.886573 |  | 2754.58 | 172.25 | 2470.176924 | 3038.728212 |
| 2028 | 1944.08 | 136.75 | 1719.560966 | 2168.685667 |  | 2751.08 | 209.02 | 2405.94624 | 3095.918396 |
| 2029 | 1947.64 | 161.67 | 1682.206555 | 2213.164637 |  | 2747.65 | 248.11 | 2337.94993 | 3157.012518 |
| 2030 | 1951.35 | 188.08 | 1642.549259 | 2260.253432 |  | 2744.35 | 289.38 | 2266.502773 | 3221.823102 |
| 2031 | 1955.27 | 215.94 | 1600.723433 | 2309.933421 |  | 2741.16 | 332.7 | 2191.767439 | 3290.12486 |
| 2032 | 1959.37 | 245.22 | 1556.765127 | 2362.107515 |  | 2737.98 | 377.97 | 2113.832482 | 3361.673455 |
| 2033 | 1963.52 | 275.82 | 1510.671695 | 2416.525579 |  | 2734.83 | 425.08 | 2032.886631 | 3436.285983 |
| 2034 | 1967.62 | 307.69 | 1462.437215 | 2472.969481 |  | 2731.72 | 473.93 | 1949.090271 | 3513.820604 |
| 2035 | 1971.67 | 340.8 | 1412.123574 | 2531.381868 |  | 2728.7 | 524.48 | 1862.604398 | 3594.232437 |
| 2036 | 1975.7 | 375.13 | 1359.80463 | 2591.791314 |  | 2725.88 | 576.68 | 1773.576975 | 3677.56564 |
